# Whole‐Genome Insights Into Tigecycline‐Resistant *Klebsiella* Spp. Harboring Colistin Resistance Gene From Food Animals in Türkiye

**DOI:** 10.1155/tbed/9122655

**Published:** 2026-04-20

**Authors:** Cemil Kürekci, Xiaoyu Lu, Seyda Şahin, Yeşim Soyer, Zhiqiang Wang, Jens A. Hammerl, Ruichao Li

**Affiliations:** ^1^ Department of Food Hygiene and Technology, Faculty of Veterinary Medicine, Hatay Mustafa Kemal University, Hatay, Türkiye, mku.edu.tr; ^2^ Department of Microbiology, Faculty of Veterinary Medicine, Dokuz Eylül University, Izmir, Türkiye, deu.edu.tr; ^3^ Jiangsu Co-Innovation Center for Prevention and Control of Important Animal Infectious Diseases and Zoonoses, College of Veterinary Medicine, Yangzhou University, Yangzhou, Jiangsu Province, China, yzu.edu.cn; ^4^ College of Pharmacy, Taizhou University, Taizhou, Jiangsu Province, China, tzc.edu.cn; ^5^ Department of Food Hygiene and Technology, Faculty of Veterinary Medicine, Sivas Cumhuriyet University, Sivas, Türkiye, cumhuriyet.edu.tr; ^6^ Department of Food Engineering, Middle East Technical University, Ankara, Türkiye, metu.edu.tr; ^7^ Department Biological Safety, German Federal Institute for Risk Assessment, Berlin, Germany, bfr.bund.de

**Keywords:** *Klebsiella pneumoniae*, *mcr*-8, tigecycline resistance, whole-genome sequencing

## Abstract

Antimicrobial‐resistant (AMR) *Klebsiella* spp., the common agents of nosocomial infections, are on the rise and becoming increasingly dangerous, with tigecycline‐resistant strains posing a serious clinical threat. This study aimed to characterize isolated tigecycline‐resistant strains from Türkiye by using whole‐genome sequencing (WGS). The genomes of 40 strains, recovered from turkey caecum samples (*n* = 23, *K. pneumoniae*), chicken caecum samples (*n* = 8, *K. pneumoniae*; *n* = 2, *K. oxytoca*), and chicken meat samples (*n* = 7, *K. pneumoniae*), were determined. Additionally, Oxford Nanopore Sequencing was conducted on subsets (*mcr*‐8‐positive, *n* = 9) to characterize the plasmids responsible for colistin resistance spread. Sequence analysis revealed that all *K. pneumoniae* harbored the *tet*(A) gene without detectable mutations, whereas several alterations were detected in the *ramR* gene. Additionally, other tetracycline‐encoding genes were also detected in few isolates, including *tet*(B), *tet*(D), *tet*(J), and *tet*(M). Genomic analysis revealed various AMR genes, including the plasmid‐mediated colistin resistance gene *mcr*‐8, for which hybrid assemblies confirmed the localization on IncF group plasmids associated with IS*903B*. Conjugation assay showed the transferability of *mcr*‐8 in isolates (8/9) to *Escherichia coli* recipient strains. WGS analysis also revealed 11 different sequence types (STs), with ST37 appearing as the predominant lineage among both tigecycline‐ and colistin‐resistant isolates. Phylogenetic comparison with previously sequenced isolates showed close genetic relatedness between certain isolates and international clones. To our knowledge, this is the first report of *mcr*‐8 in *Klebsiella* strains from food animals in Türkiye, highlighting a potential risk for transmission of last‐resort antimicrobial resistance genes to humans. Hence, continuous genomic surveillance is urgently needed to monitor the spread of these resistance determinants within the One Health perspective.

## 1. Introduction

*Klebsiella pneumoniae* (*K. pneumoniae*), a Gram‐negative bacterium, is considered one of the most important opportunistic pathogens in nosocomial and community infections such as septicemia, urinary tract infection, and pneumonia, which can be fatal to immunocompromised patients [1]. Given the inherent resistance of *K. pneumoniae* to a wide array of antimicrobials driven by chromosomally encoded mechanisms such as the β‐lactamase gene (*bla*_SHV_), the fosfomycin resistance gene (*fosA*), and the nalidixic acid efflux pump encoding the *oqxAB* gene, the clinical management of its infections poses a considerable challenge [2, 3]. Recently, acquisition of carbapenem resistance in clinical *K. pneumoniae* strains is classified as a “critical concern” because of hampered treatment due to resistance development [4]. Tigecycline and colistin are the last‐line of therapeutic options for the treatment of carbapenem‐resistant *K. pneumoniae* [5].

Tigecycline is a semi‐synthetic derivative of minocycline antibiotic that exhibit antimicrobial activity toward a broad spectrum of pathogenic bacteria; however, utilization of these antibiotics has resulted in an increase of resistance. Tigecycline resistance is commonly linked to an active drug efflux system and enzymatic degradation, which have been frequently linked with mobile genetic elements among the strains of *Escherichia coli* and *K. pneumoniae* [6]. Several types of resistance genes have been found to be important for tigecycline resistance including *ramR*, *oqxR*, *rpsj*, *tet*(A), *tet*(L), *tet*(M), and *tet*(X) [6, 7]. *tet*(A) mutant types were first identified among a population of *K. pneumoniae* [7], and subsequent *tet*(A) mutants have been reported [8, 9]. Mutation in *ramR* leads to de‐repression of *ramA*, which upregulates the expression of the AcrAB‐TolC efflux pump genes, leading to increased efflux pump production and enhanced tigecycline resistance [7, 10]. Most importantly, tigecycline resistance has, however, been linked to various *tet*(X) gene variants encoding FAD‐dependent monooxygenase, the most common of which is *tet*(X4) [5]. Additional plasmid‐mediated resistance‐nodulation‐division (RND)‐type efflux pump gene cluster, *tmexCD-toprJ*, has been recently described in tigecycline‐resistant *K. pneumoniae* [11, 12].

Recently, tigecycline‐resistant *K. pneumoniae* strains harboring *tet*(X4) and *tmexCD-toprJ* variants have been isolated from food, clinical, and environmental isolates in different countries, especially in China [13–16]. Additionally, it became evident that *K. pneumoniae* originating from food animals with multiple resistance have been built up, and co‐resistance of colistin, frequently associated with *mcr* genes, and tigecycline had been documented [17]. However, information related to their frequencies in animal production in Türkiye remains scarce.

In addition to the importance of tigecycline resistance and its underlying mechanisms, colistin resistance in *K. pneumoniae* represents a major global public health concern as colistin is also considered a last‐resort antimicrobial for the treatment of infections caused by multidrug‐resistant, including carbapenem‐resistant *Enterobacterales*. The emergence of colistin resistance severely limits therapeutic options and has been associated with increased mortality rates in humans. Of particular concern is the emergence of plasmid‐mediated mobile colistin resistance (*mcr*) genes (*mcr*‐1 to *mcr*‐10), which can be readily transferred between bacterial species via plasmid conjugation, facilitating their rapid global dissemination [18]. While *mcr*‐1 is the most prevalent and extensively studied variant, epidemiological investigation of less common *mcr* genes, such as *mcr*‐8, may provide valuable insights into the dissemination dynamics and transmission routes of these resistance determinants, particularly at the human–animal interface [19, 20].

A large‐scale study, in which caecal samples from chickens and turkey, alongside retail meat (chicken, beef, and mutton), were screened for tigecycline‐resistant *Enterobacterales*, documented *tet*(X4)‐carrying *Escherichia coli* in 4.6% of chicken caeca, 8.17% of retail chicken meat, and 0.6% of red meat samples [21]. However, all isolated tigecycline‐resistant *Klebsiella* spp. strains, defined as having an MIC of >0.5 µg/mL (as the cut‐off point of EUCAST), were negative for *tet*(X4) and *tmexCD-toprJ* [21]. Therefore, the present work aimed to characterize these tigecycline‐resistant *Klebsiella* spp. by short‐read (Illumina) and long‐read (Oxford Nanopore Technologies) sequencing and to decipher the underlying genomic characterization of such strains and global resistant strains.

## 2. Materials and Methods

### 2.1. Bacterial Isolates

A total of 40 *Klebsiella* spp. isolates from turkey caecum samples (*n* = 23, *K. pneumoniae*), chicken caecum samples (*n* = 8, *K. pneumoniae*; *n* = 2, *K. oxytoca*), and chicken meat samples (*n* = 7, *K. pneumoniae*) with the MIC of tigecycline ranging from 1 µg/mL to 8 µg/mL were selected for WGS. The isolates were chosen based on the sampling source and antimicrobial resistance profile.

### 2.2. Genome Sequencing and Analysis

Genomic DNAs of isolates were extracted using the PureLinkTM Genomic DNA mini kit (Invitrogen) according to the manufacturer’s protocol and quantified by a Qubit 4 Fluorometer (Invitrogen). Then, samples were used for library construction and sequencing using the Illumina HiSeq 2500 platform, resulting in 150 bp paired‐end reads. SPAdes version 3.14.0 [22] was used for *de novo* assembling of the short‐read sequences. The draft genomic assembly was curated using prokka [23]. Based on the presence of the *mcr*‐8 gene, nine isolates were further selected for long‐read Oxford Nanopore Technology (ONT) sequencing. Complete genome sequences were obtained using Unicycler with default parameters [24]. The antibiotic resistance genes, plasmid replicon types, and insertion sequences were retrieved by ResFinder (v4.0), PlasmidFinder (v2.0), and ISfinder, respectively [25–27]. BRIG and Easyfig were used to display the plasmid organization and gene environment features, respectively [28, 29].

Mutation profiling of the *ramR* and *tet*(A) genes was performed by screening for sequence variations using the reference genome of *K. pneumoniae* ATCC 43816 and the sequence (GenBank, accession number AJ517790) with BLASTp‐based comparative alignments.

### 2.3. Phylogenetic Relationships

To obtain a more comprehensive understanding, we downloaded all available *mcr*‐8‐positive *K. pneumoniae* genomes from the database for comparative analysis. A total of 194 genomes of *K. pneumoniae* isolates carrying *mcr*‐8 were retrieved from the NCBI database (May 15, 2025) using the following search criteria “AMR_genotypes:mcr‐8*” and then were downloaded using the NCBI datasets tool (https://www.ncbi.nlm.nih.gov/datasets/) according to the GenBank assembly number (GCA_*). The MLST tool was used to determine the multilocus sequence type (MLST) of the isolates. Then, phylogenetic analysis using Roary and FastTree [26] was applied based on single nucleotide polymorphisms (SNPs) of core genomes [30, 31] https://github.com/tseemann/snp-dists). The phylogenetic tree was visualized and annotated using the online tool iTOL [32].

### 2.4. Conjugation Assay

Conjugation assays were carried out using the liquid mating approach with *E. coli* C600 (rifampicin‐resistant) serving as a recipient for plasmid transmission. Briefly, overnight cultures of *E. coli* C600 and *K. pneumoniae* isolates carrying *mcr*‐8 were mixed in equal proportion (1/1; v/v) and incubated onto LB agar plates at 37°C for 20 h. Following incubation, the conjugation mixture was taken into sterile saline. After centrifugation and processing, the mixture was spread onto LB agar plates supplemented with 2 µg/mL colistin and 300 µg/mL rifampin. Double‐resistant colonies (*E. coli*) were retained as transconjugants. The presence of *mcr*‐8 in transconjugants was confirmed by PCR and the corresponding resistance phenotypes.

## 3. Results

### 3.1. Genomic Characteristics of *Klebsiella* spp. Isolates

**Figure 1:**
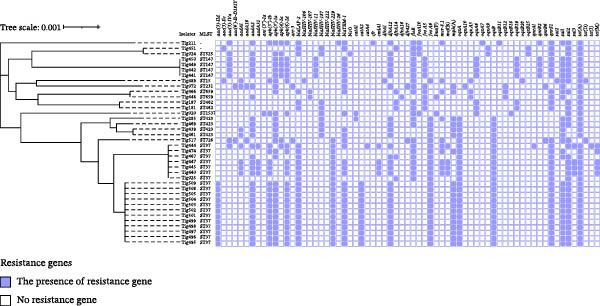
Phylogenetic relationship of the tigecycline‐resistant *K. pneumoniae* strains. The MLST types and AMR‐encoding genes are shown.

Among 40 *K. pneumoniae* genomes, 67 antibiotic resistance genes were detected. All tigecycline‐resistant *K. pneumoniae* (except one) harbored *tet*(A), as well as other determinants like *tet*(M) (*n* = 5), *tet*(D) (*n* = 2), and *tet*(J) (*n* = 1) (Figure 1 and Supporting Information 2: Table S1). When compared to wild‐type *tet*(A), there was no mutation detected in the genes of our isolates. However, we observed eight distinct mutations in the *ramR* gene of 40 isolates (Supporting Information 1: Figure S1), including substitutions and alterations distributed throughout the coding region (Supporting Information 1: Figure S2).

Almost all strains (*n* = 38) carry the *fosA* gene variants (*fosA5*, *fosA6*, and *fosA10*), which is associated with fosfomycin resistance. Beta‐lactamase genes, like *bla*_SHV-_variants, *bla*_TEM−1_ and *bla*_LAP−2_ were also prevalent, while the *bla*_OXY−2_ gene (*n* = 2) and *bla*_TEM−176_ (*n* = 1) were detected in only *K. oxytoca* strains (Figure 1 and Supporting Information 2: Table S1).

The following resistance genes were also found: aminoglycoside resistance genes [*aadA*1 (*n* = 10), *aadA*2 (*n* = 29), *aadA*16 (*n* = 3), *aadA*31 (*n* = 4), *aadA*5 (*n* = 1), *aph*(3^′^’)‐Ia (*n* = 31), *aph*(3^′^’)‐Ib (*n* = 21), *aph*(4)‐Ia (*n* = 8), *aph*(6)‐Id (*n* = 25), *ant*(3^′^’)‐Ia (*n* = 1), *aac*(3)‐IIe (*n* = 3), *aac*(3)‐IId (*n* = 12), *aac*(3)‐IVa (*n* = 8), *aac*(6^′^)Ib‐D181Y (*n* = 3)], sulfonamide resistance genes [*sul*1 (*n* = 30), *sul*2 (*n* = 33), and *sul*3 (*n* = 5)], quinolone resistance genes [*oqxA/B* (*n* = 40), *qnrS* (*n* = 32), *qnrB*2 (*n* = 5), *qnrB*6 (*n* = 2), and *qnrA*1 (*n* = 1)], trimethoprim resistance genes [*dfrA*12 (*n* = 28), *dfrA*14 (*n* = 7), *dfrA*16 (*n* = 1), *dfrA*1(*n* = 1), *dfrA*17 (*n* = 1), *dfrA*27 (*n* = 3)], phenicol resistance genes [*floR* (*n* = 25), *catA*1 (*n* = 3), *catA*2 (*n* = 12), *catA*4 (*n* = 1), *cfr* (*n* = 1), *cmlA*1 (*n* = 4)] lincosamide resistance gene [*lnu*(F) (*n* = 3)], rifampicin resistance gene [*arr*−3 (*n* = 3)], macrolide resistance genes [*mph*(A) (*n* = 21) and *mef*(B) (*n* = 4)] and streptomycin resistance genes [*strA* (*n* = 20) and *strB* (*n* = 24)] (Figure 1 and Supporting Information 2: Table S1). Most importantly, *Klebsiella* spp. strains [*K. pneumoniae* (*n* = 10) and *K. oxytoca* (*n* = 1)] displayed the presence of *mcr*‐8, which confers resistance to colistin.

### 3.2. Characterization in *mcr*‐8‐Positive Plasmids

**Figure 2:**
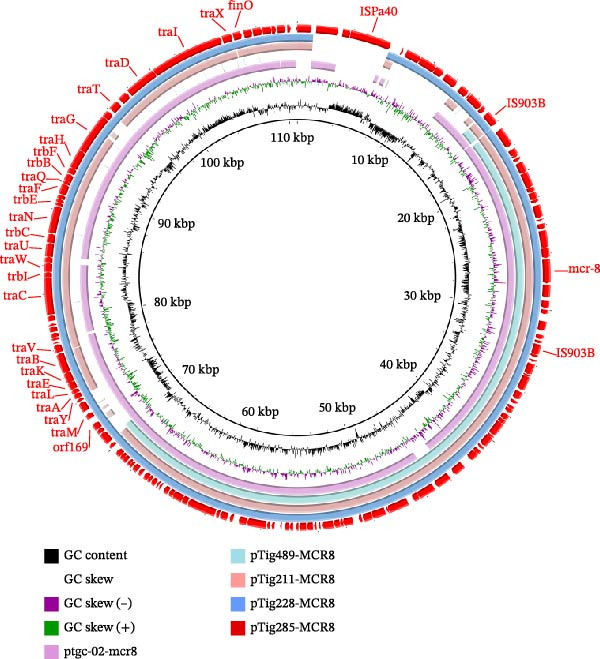
Circular comparisons among *mcr*‐8‐bearing plasmids with repB(R1701)‐type plasmid structure. The outmost circle indicates the plasmid pTig440‐MCR8. Selected genes associated with plasmid transfer, resistance, etc. are indicated.

In isolates Tig285 and Tig228, the *mcr*‐8 genes were located on the IncFII(K)‐repB(R1701) plasmids pTig285‐MCR8 and pTig228‐MCR8. In isolate Tig211, the *mcr*‐8 gene was located on the IncFII(pKP91)‐repB(R1701) plasmid pTig211‐MCR8. In isolate Tig489, *mcr*‐8 was located on the repB(R1701) plasmid pTig489‐MCR8. No other resistance gene was found on these plasmids. BLASTn search revealed that pTig285‐MCR8 and pTig228‐MCR8 exhibited high homology to the plasmid ptgc‐02‐mcr8 (CP132217) in the *K. pneumoniae* strain from human with >98.96% identity at 89% coverage. Plasmid pTig211‐MCR8 showed 98.92% identity at 87% coverage with plasmid ptgc‐02‐mcr8. pTig489‐MCR8 shared 98.83% identity at 97% coverage with plasmid ptgc‐02‐mcr8 (Figure 2). ptgc‐02‐mcr8 was an IncFII(K)‐repB(R1701)‐IncQ1 hybrid plasmid. The *mcr*‐8 in all these isolates was located on the repB(R1701)‐type plasmid structure region. The repB(R1701)‐derived plasmids identified here exhibit high homology with plasmids from human clinical strains, suggesting that *mcr*‐8‐positive repB(R1701) plasmids may have facilitated cross‐species transmission between animal‐derived and human‐derived bacteria, thereby potentially posing a public health risk. With the exception of pTig489‐MCR8, all other plasmids carry type IV secretion system genes and possess conjugation transferability, indicating that repB(R1701)‐plasmids can acquire horizontal transfer capability through fusion with other conjugative plasmids, which may accelerate the dissemination of the *mcr*‐8 gene among *Enterobacterales*. Plasmid structure comparisons reveal that repB(R1701)‐plasmids can form hybrid plasmids through modular recombination (e.g., fusion with other plasmids, such as IncFII/IncQ1‐type plasmids). This genetic plasticity may promote the evolution of multidrug resistance, necessitating continuous monitoring of the structural variation trends of such plasmids.

In isolates Tig440, Tig444, and Tig517, *mcr*‐8 genes were located on the IncFIA(HI1) plasmids pTig440‐MCR8, pTig444‐MCR8, and pTig517‐MCR8. In isolate Tig474, *mcr*‐8 was located on the IncFIA(HI1)‐IncFII(pKP91) plasmid pTig474‐MCR8. No other resistance gene was found in these plasmids. Sequence analysis revealed that pTig440‐MCR8 shared 99.45% identity at 95% coverage with plasmid pSYCC2_mcr‐8_103k (CP113187) in *K. pneumoniae* isolated from chicken and 98.21% identity at 95% coverage with plasmid pKP19‐2581_101k_mcr8 (CP120873) in *K. pneumoniae* isolated from human. Additionally, it exhibited 98.50% identity at 90% coverage with plasmid p2018C01‐0461_MCR8 (CP044369) in *K. pneumoniae* isolated from human (Figure 3A). pTig474‐MCR8 showed 97.92% identity at 56% coverage with plasmid p2018C01‐046‐1_MCR8, which was also an IncFIA(HI1)‐IncFII(pKP91) plasmid. Isolates Tig440, Tig445, and Tig447 possessed the same plasmid replicons and resistance genes, and they were classified as the same ST, leading to hypothesize that the *mcr*‐8‐positive plasmids they carried were similar. Through comparing the draft assembly sequences of Tig445 and Tig447 with plasmid pTig440‐MCR8, it was found that there was a high degree of identity and coverage, indicating that *mcr*‐8 was also located on the IncFIA(HI1) plasmid in isolates Tig445 and Tig447 (Figure 3B). The *mcr*‐8 in all these isolates was located on the IncFIA(HI1)‐type plasmid structure region, and they possessed type IV secretion system genes that is responsible for the conjugative transfer of plasmids. The consistent presence of a functional type IV secretion system across all IncFIA(HI1)‐linked *mcr*‐8‐positive plasmids indicates an inherent potential for horizontal transfer, which could accelerate the dissemination of colistin resistance among *Enterobacterales* in diverse ecological niches. The coexistence of identical ST types and closely related plasmid structures in multiple isolates (Tig440, Tig445, and Tig447) implies concurrent clonal transmission and plasmid‐mediated horizontal dissemination, a dual mechanism that may enhance the persistence and propagation of *mcr*‐8 in bacterial populations. The identification of an IncFIA(HI1)‐IncFII(pKP91) hybrid plasmid (pTig474‐MCR8) underscores the recombinogenic nature of these vectors. Such modular rearrangements may broaden the host range or stabilize plasmid maintenance, further complicating containment of *mcr*‐8 dissemination.

Figure 3Circular comparisons among *mcr*‐8‐bearing plasmids with IncFIA(HI1)‐type plasmid structure (A, B). The outmost circle indicates the plasmid pTig440‐MCR8. Genes associated with important functions are indicated.

**Figure:**
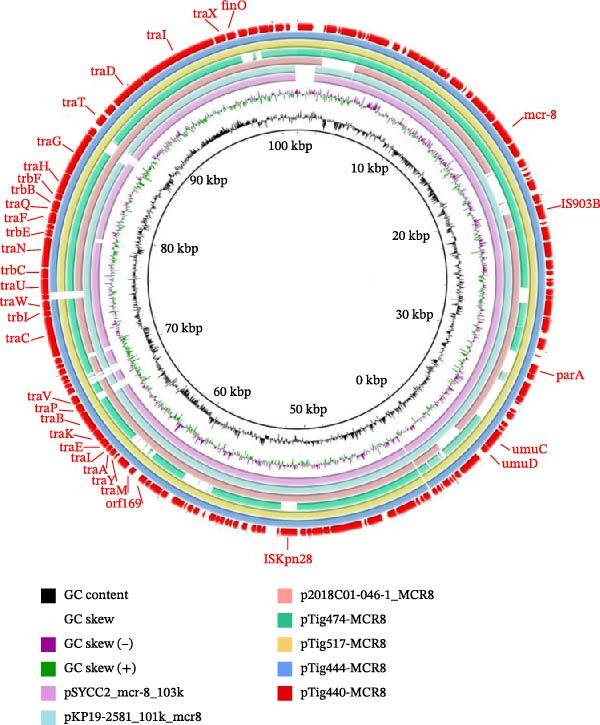
(A)

**Figure:**
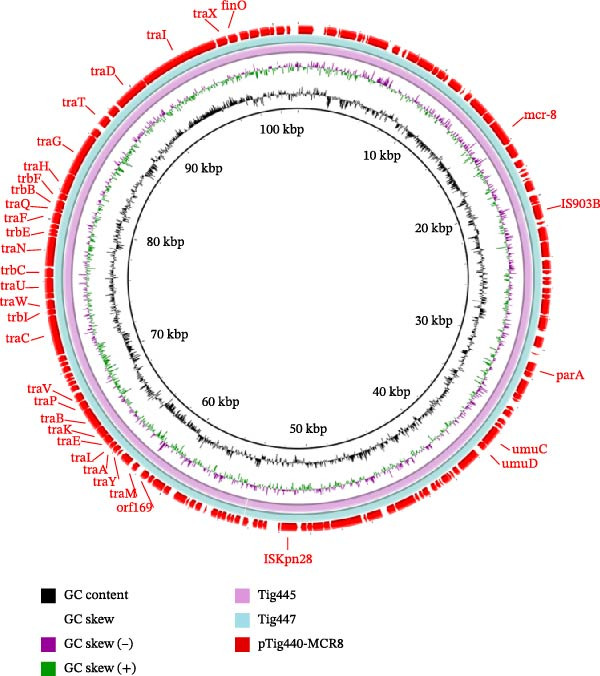
(B)

**Figure 4:**
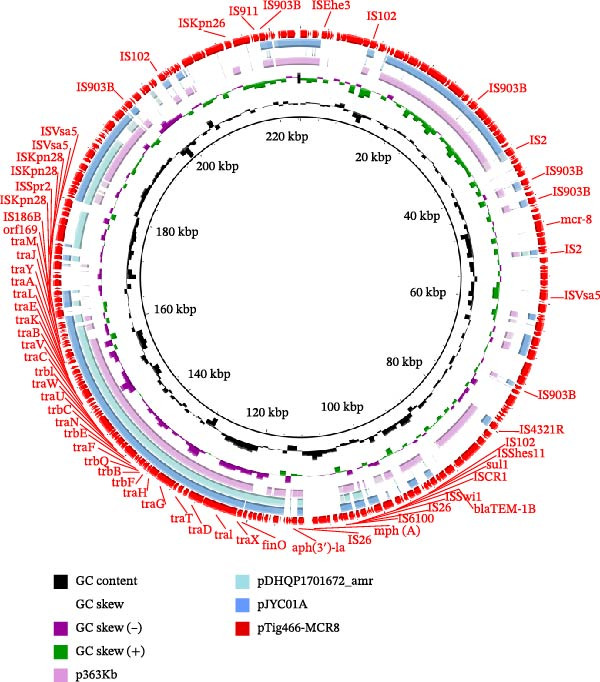
Circular comparisons between *mcr*‐8‐bearing IncFIB(K)‐IncFII(K) plasmid pTig466‐MCR8 and other similar plasmids in the NCBI nr database. The outmost circle indicates the plasmid pTig440‐MCR8. Genes of interest are indicated.

**Figure 5:**
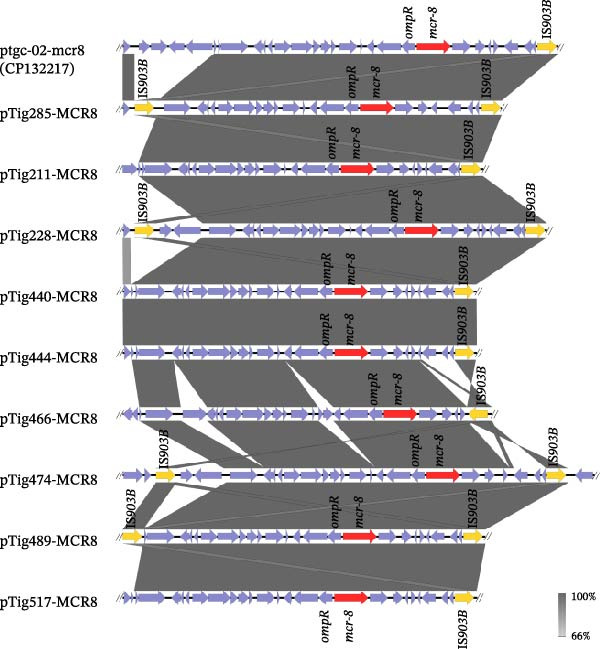
Genetic context structure of *mcr*‐8 gene region with closely related sequence (CP132217).

In isolate Tig466, *mcr*‐8 was located on the IncFIB(K)‐IncFII(K) plasmid pTig466‐MCR8. It is a multidrug‐resistant plasmid carrying various resistance genes, including *aph*(*3^′^*)*-Ia*, *bla*_TEM−1 B_, *mcr*‐8, *mph*(A), and *sul1*. BLASTn search revealed that pTig466‐MCR8 exhibited >99% identity at >50% coverage with plasmid pJYC01A (CP022926) and plasmid p363Kb (CP095777) in *K. pneumoniae* isolated from human. It also shared 98.78% identity at 31% coverage with plasmid pDHQP1701672_amr (CP037744) in *K. pneumoniae* isolated from human (Figure 4). This plasmid, pTig466‐MCR8, features a relatively novel structure and carries multiple classes of antibiotic resistance genes, leading to a “cumulative effect” of resistance determinants. This may facilitate the emergence of multidrug‐resistant phenotypes, substantially increasing the clinical treatment burden. Moreover, its possession of type IV secretion system‐related genes confers conjugative transfer capability, indicating a practical ability for horizontal transmission between animal‐derived and human‐derived bacteria. Consequently, it significantly raises the risk of multidrug resistance dissemination within clinical and environmental bacterial populations. The findings also indicated a close association between the *mcr-8* gene and IS903B; however, the configuration of these elements appeared to vary (Figure 5).

### 3.3. The Transferability of the *mcr*‐8 Plasmids and Plasmid Replicon Types

The conjugation assay also confirmed the transferability of the *mcr*‐8 plasmid in eight isolates (except Tig489). In this study, 19 different plasmid replicons were determined among all *Klebsiella* spp. strains, with ColRNAI, IncFIA(HI1), IncFIB(K), IncFII(pCRY), IncFII(pKP91), and IncR being the most frequently detected ones (Supporting Information 3: Table S2). Several insertion sequences (IS) were found in *Klebsiella* spp. strains are listed in Supporting Information 4: Table S3.

### 3.4. MLST Analysis

A total of 11 different sequence types (STs) were detected, including ST37 (*n* = 19), ST147 (*n* = 4), ST423 (*n* = 4), ST462 (*n* = 2), ST659 (*n* = 2), ST15 (*n* = 1), ST231 (*n* = 1), ST525 (*n* = 1), ST726 (*n* = 1), and ST1537 (*n* = 1). ST37 and ST423 were the only sequence types originated from turkey and chicken caecum, and chicken meat samples. ST147 originated from only turkey caecum samples (Figure 1). Two isolates were obtained with unknown ST types. *K. oxytoca* (*n* = 2) was identified as ST145.

### 3.5. Comparative Phylogenetic Analysis of *mcr*‐8‐Positive Isolates With Global *mcr*‐8 Strains

**Figure 6:**
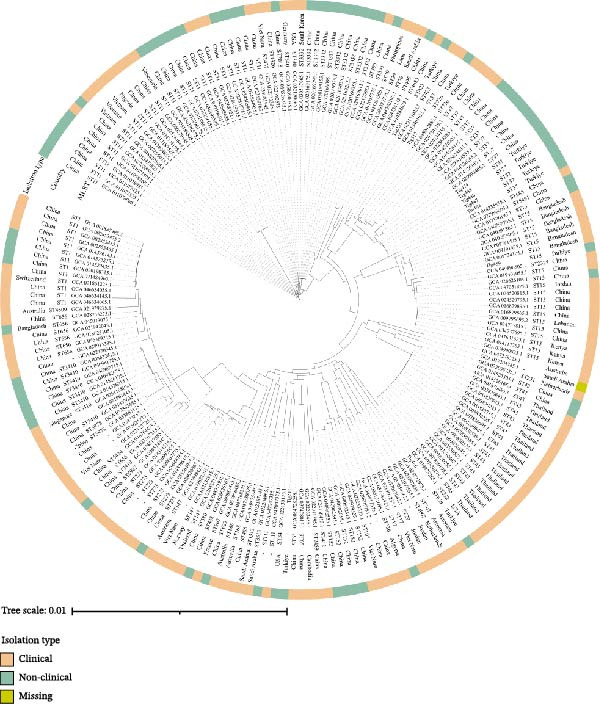
Phylogenetic relationship of *mcr*‐8‐positive *K. pneumoniae* strains in this study and those obtained from NCBI. ST type, country and isolation source are indicated in the figure.

Phylogenetic analysis demonstrated that the *mcr*‐8 carrying *K. pneumoniae* isolates from Türkiye were distributed among several distinct clades, reflecting considerable genetic heterogeneity. Importantly, multiple Turkish isolates clustered closely with internationally reported strains, particularly from China, within ST15, ST37, ST726, and ST423 lineages (Figure 6). The short branch lengths observed in these clusters indicate a high level of genomic relatedness and suggest recent common ancestry, supporting the possibility of transboundary dissemination of these resistance determinants.

## 4. Discussion

Currently, tigecycline and colistin are used as the last‐line therapeutic options for the treatment of infections caused by multidrug‐resistant Gram‐negative bacteria [33]. In clinical *K. pneumoniae*, resistances to tigecycline have been reported to be uncommon but increasingly occur in food‐producing animals representing a clinical concern in future human infection management [9]. Hence, this study undertook comparative exploration of tigecycline‐resistant *K. pneumoniae* strains previously recovered from the avian species and chicken meat samples [21].

Previous studies have shown that tigecycline resistance in *K. pneumoniae* is frequently associated with the overexpression of efflux pumps such as AcrAB and OqxAB, as well as mutations in the *tet*(A) gene, which encodes the Tet(A) efflux protein [6, 9, 34, 35]. In a study conducted by Chiu et al. [7], it was demonstrated that an 8‐fold increase in resistance to tigecycline resulted from type I and type II *tet*(A). Peng et al. [9] also noted that type III caused a 4‐fold increase. Additionally, plasmid‐mediated multidrug resistances (MDR) including *tet*(X4) and *tmexCD-toprJ* variants [17, 36] have also been identified in tigecycline‐resistant *K. pneumoniae*. Consistent with our previous findings through PCR assays [21], WGS analysis, however, showed that tested strains did not have neither these genes nor mutation in the *tet*(A) gene when compared with the reference sequence. Notably, consistent with our findings, Li et al. [36] reported *K. pneumoniae* recovered from hospital wastewater in China with tigecycline MICs (8–16 μg/mL), in which known resistance determinants could not be identified, suggesting the existence of novel or uncharacterized resistance mechanisms. Our analysis, however, showed that the efflux pump genes *oqxA* and *oqxB* were common among the isolates. This RND‐type pump has been shown to decrease intracellular accumulation of different antibiotics including aminoglycosides, β‐lactams, macrolides, fluoroquinolones, and importantly tigecycline, which often correlates with additional mutations in the *ramR* gene [10, 37]. Additionally, we identified eight distinct mutations within the *ramA* gene, which is known to activate the AcrAB‐TolC efflux system [38]. However, since the expression levels of this gene were not assessed in the current study, we, therefore, were unable to establish a direct involvement of these variations without functional confirmation. Further functional studies, including gene expression profiling and efflux pump activity assays, are required to elucidate the potential role of these *ramA* mutations in mediating resistance.

WGS analysis revealed that tigecycline‐resistant *Klebsiella* spp. strains carry a complex resistome including fluoroquinolones (*qnr*), sulfonamides (*sul*1/*sul*2), fosfomycin (*fosA*), and colistin (*mcr*). These findings are consistent with previous reports [17], which highlight the global trend of multidrug resistance in *K. pneumoniae* from livestock. WGS analysis also revealed that colistin‐resistant *K. pneumoniae* strains and one *K. oxytoca* strain carrying *mcr*‐8 are highly prevalent in avian livestock production, indicating significant consumer exposure risk. However, some studies showed the presence of the *mcr*‐1 and *mcr*‐2 genes in *E. coli* and *K. pneumoniae* isolated from foods of animal origins and human clinical samples in Türkiye [39–42]. To the best of our knowledge, this is the first report from Türkiye for the occurrence of *mcr*‐8‐positive *Klebsiella* spp. strains from animal origin.

In the current study, *mcr*‐8‐positive *K. pneumoniae* strains harbored IncFII/IncFIA/IncFIB plasmids, which mostly did not carry any other resistance gene and all carried a type IV secretion system, facilitating the dissemination. Of note, our comparative analysis revealed that these plasmids have also been reported in human clinical *K. pneumoniae* strains, highlighting their potential animal–human dissemination. Similar to previous reports [19, 43] describing the *mcr*‐8 gene localized in plasmid, where this trait was typically flanked by two oppositely oriented IS*903B* elements, our findings also revealed a close link between the *mcr*‐8 gene and IS*903B*, for which the arrangements were varied. These structural variations suggest that IS*903B* may play a versatile role in the mobilization and rearrangement of *mcr*‐8, potentially contributing to the genetic diversity of its plasmid backbones.

In the present study of avian‐related *Klebsiella* isolates, a high degree of clonal diversity was observed. Among these, ST37 was identified as the most frequent clone, while two additional lineages, ST147 and ST423, were represented by four isolates each. Notably, colistin‐resistant *mcr*‐8‐carrying isolates were also predominantly associated with ST37; other STs were present tough. The occurrence of the high‐risk *K. pneumoniae* ST37 clone has been documented in multiple hosts, including poultry, companion animals, and humans [20, 44]. The predominance of ST37 among our isolates, coupled with its frequent association with colistin resistance, suggests that this lineage may act as a significant reservoir, which further requires genomic and epidemiological investigations to monitor its public health significance. Additionally, MDR hypervirulent *K. pneumoniae* ST147 isolates from human clinical settings have been identified worldwide, raising serious concerns [44, 45]. Importantly, one *mcr*‐8 positive isolate belonged to ST15, which was described as clinically relevant lineage by previous studies [8, 46].

Although xthis study provides an in‐depth genomic analysis, several limitations should be acknowledged. The relatively limited number of tigecycline‐resistant *Klebsiella* isolates may impact the broader applicability of the results. In addition, the lack of parallel analysis of clinical isolates restricts more definitive conclusions regarding transmission between the food‐animal reservoir and human healthcare settings. Finally, the stability, transmissibility, and fitness impact of the *mcr*‐8‐harboring plasmids were not addressed here and warrant further functional investigation.

## 5. Conclusion

This study provides the first genomic characterization of tigecycline‐resistant *Klebsiella* spp. strains isolated from chicken and turkey as well as chicken meat in Türkiye. Whole‐genome sequencing revealed the widespread distribution of wild‐type *tet*(A) in all *K. pneumoniae* strains and eight distinct mutations in the *ramR* gene among all *Klebsiella* spp. strains. Although direct transmission links could not be inferred from this study, the finding of the *mcr*‐8 gene localized on IS903B‐associated IncF plasmids in *K. pneumoniae* isolates from food animals underscores the significant potential for these mobile resistance determinants to spread within the broader One Health framework.

## Author Contributions

Cemil Kurekci, Seyda Şahin, and Yeşim Soyer collected samples and isolated bacteria. Cemil Kurekci and Xiaoyu Lu performed the experiments, analyzed the data, and drafted the manuscript. Zhiqiang Wang, Jens A. Hammerl, and Ruichao Li assisted in the data analysis. All authors edited, read, and approved the final manuscript. The project was supervised by Cemil Kurekci and Ruichao Li.

## Funding

The present work was supported by the Scientific and Technological Research Council of Turkiye (TUBITAK, Project No: 121N855), the National Natural Science Foundation of China (no. 32161133005), and the Priority Academic Program Development of Jiangsu Higher Education Institutions (PAPD).

## Ethics Statement

Ethical approval was not required for this study, as the isolates analyzed were part of a previously established strain collection obtained from deceased animals during routine slaughterhouse procedures and described in an earlier publication.

## Conflicts of Interest

All authors have no conflicts of interest or financial ties to disclose.

## Supporting Information

Additional supporting information can be found online in the Supporting Information section.

## Supporting information


**Supporting Information 1** Figure S1: ramR nucleotides sequence with indicated mutations. Figure S2: RamR amino acid sequence.

**Supporting Information 2** Table S1: Antimicrobial resistance genes of tigecycline‐resistant *Klebsiella spp*. isolates.

**Supporting Information 3** Table S2: Replicon types of tigecycline‐resistant *Klebsiella spp*. isolates.

**Supporting Information 4** Table S3: IS elements of tigecycline‐resistant *Klebsiella spp*. isolates.

## Data Availability

All genomic sequences generated in this study have been deposited in the NCBI GenBank under the BioProject accession number PRJNA1201785. The full‐length sequence of plasmids pTKKO228‐MCR8, pTKKP211‐MCR8, pTKKP285‐MCR8, pTKKP440‐MCR8, pTKKP444‐MCR8, pTKKP466‐MCR8, pTKKP474‐MCR8, pTKKP489‐MCR8, and pTKKP517‐MCR8 were deposited in the GenBank database under accession no. PQ839733, PQ839734, PQ839736, PQ839737, PQ839738, PQ839739, PQ839740, PQ839741, and PQ839742, respectively.
