# Supplementary material for: Whole‐Genome Insights Into Tigecycline‐Resistant Klebsiella Spp. Harboring Colistin Resistance Gene From Food Animals in Türkiye
Source: Transbound Emerg Dis. 2026 Apr 20;2026:9122655. doi: 10.1155/tbed/9122655 (PMC13096690; doi:10.1155/tbed/9122655)
Supplement: Supplementary file 1 — Supporting Information 1 Figure S1: ramR nucleotides sequence with indicated mutations. Figure S2: RamR amino acid sequence. [file TBED-2026-9122655-s001.docx]

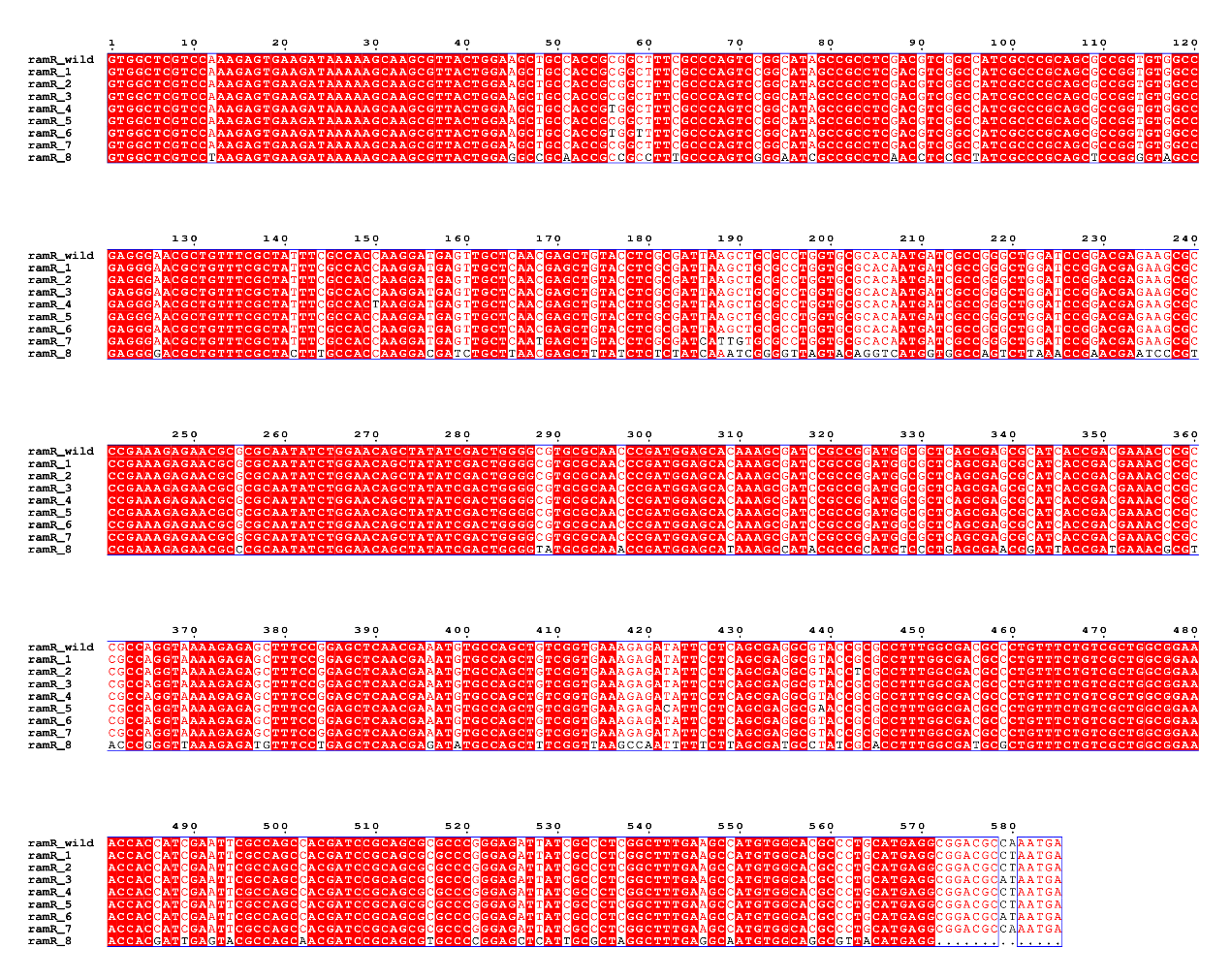
Figure S1. *ramR* nucleotides sequence with indicated mutations.


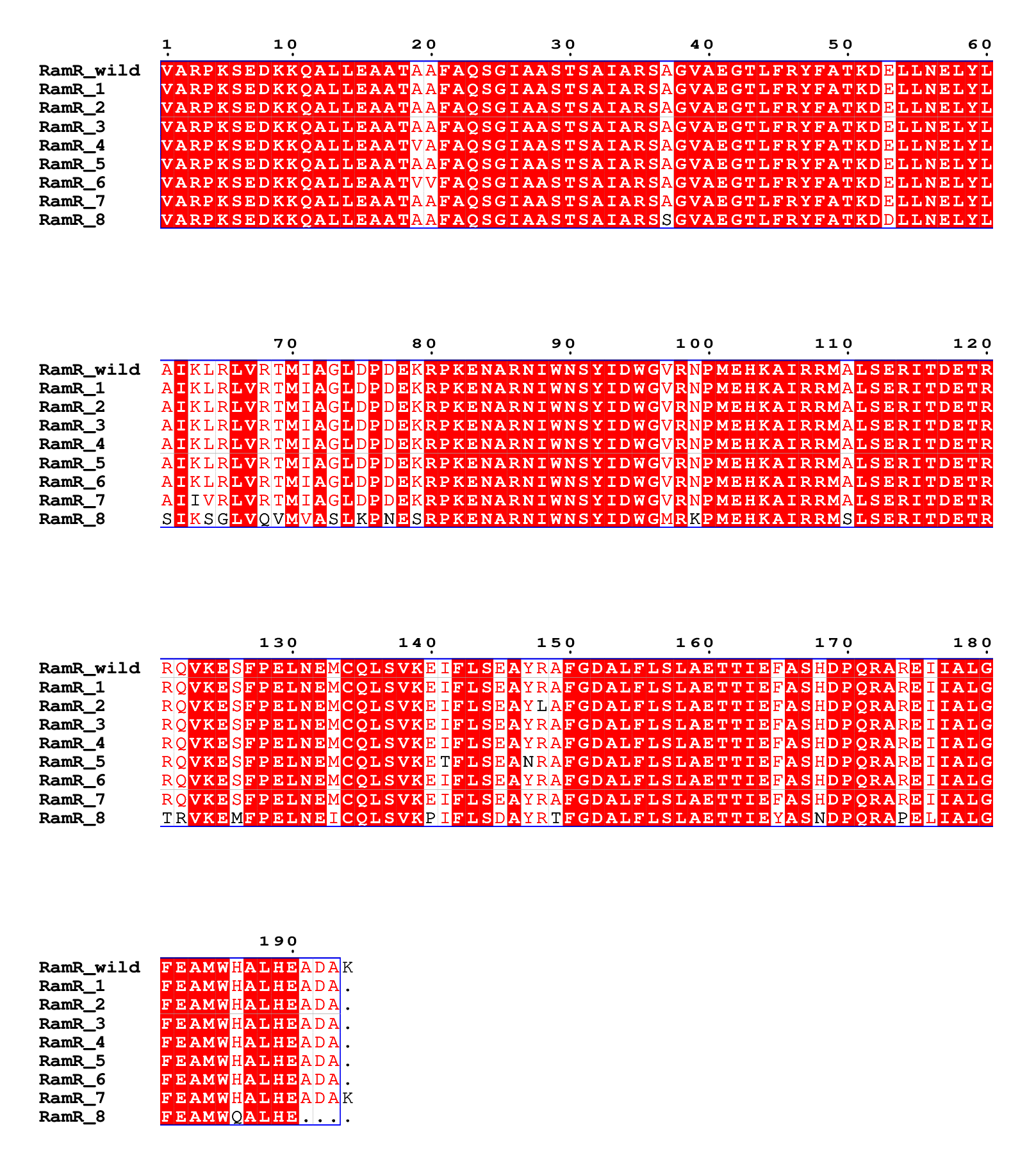
Figure S2. RamR amino acid sequence.
